# Nonintrusive and Effective Volume Reconstruction Model of Swimming Sturgeon Based on RGB-D Sensor

**DOI:** 10.3390/s24155037

**Published:** 2024-08-03

**Authors:** Kai Lin, Shiyu Zhang, Junjie Hu, Hongsong Li, Wenzhong Guo, Hongxia Hu

**Affiliations:** 1 Fisheries Science Institute, Beijing Academy of Agriculture and Forestry Sciences & National Engineering Research Center for Freshwaters (Beijing), Beijing 100068, China; linkai_bit@126.com (K.L.);; 2 Key Laboratory of Equipment and Informatization in Environment Controlled Agriculture, Ministry of Agriculture and Rural Affairs, China; 3 School of Instrument Science and Opto Electronics Engineering, Beijing Information Science and Technology University, Beijing 100192, China; 4 School of Computer Science and Technology, Beijing Institute of Technology, Beijing 100081, China; lihongsong@bit.edu.cn; 5 Intelligent Equipment Technology Research Center of Beijing Academy of Agriculture and Forestry Sciences, Beijing 100097, China; guowz@nercita.org.cn

**Keywords:** sturgeon, volume reconstruction, deep learning, nonintrusive, RGB-D sensor

## Abstract

The sturgeon is an important commercial aquaculture species in China. The measurement of sturgeon mass plays a remarkable role in aquaculture management. Furthermore, the measurement of sturgeon mass serves as a key phenotype, offering crucial information for enhancing growth traits through genetic improvement. Until now, the measurement of sturgeon mass is usually conducted by manual sampling, which is work intensive and time consuming for farmers and invasive and stressful for the fish. Therefore, a noninvasive volume reconstruction model for estimating the mass of swimming sturgeon based on RGB-D sensor was proposed in this paper. The volume of individual sturgeon was reconstructed by integrating the thickness of the upper surface of the sturgeon, where the difference in depth between the surface and the bottom was used as the thickness measurement. To verify feasibility, three experimental groups were conducted, achieving prediction accuracies of 0.897, 0.861, and 0.883, which indicated that the method can obtain the reliable, accurate mass of the sturgeon. The strategy requires no special hardware or intensive calculation, and it provides a key to uncovering noncontact, high-throughput, and highly sensitive mass evaluation of sturgeon while holding potential for evaluating the mass of other cultured fishes.

## 1. Introduction

The sturgeon has become an important commercial aquaculture species in China due, in particular, to the high economic value of its caviar [1,2]. The sturgeon aquaculture industry has developed rapidly in China, and the production reached 104,300 tons in 2020, accounting for approximately 85.07% of global production [3].

The measurement of sturgeon mass plays a remarkable role in aquaculture management, particularly in elements such as growth status estimation, feeding regime management, and grading time. Furthermore, the measurement of the mass of individual sturgeon serves as a key phenotype, offering crucial information for enhancing growth traits through genetic improvement [4]. Until now, the measurement of sturgeon mass has been usually conducted by manual sampling, which is work intensive and time consuming for farmers and invasive and stressful for the fish [5]. Therefore, nonintrusive methods for estimating sturgeon mass must be developed, a subject which has not yet been thoroughly explored in the literature.

Nonintrusive mass estimation methods can be divided into two main categories: acoustic echo methods and machine vision methods [6,7]. Acoustic echo techniques have been implemented to estimate the distribution and abundance of fish in marine and fresh-water environments, such as reservoirs and mariculture farms, while the accuracy drops substantially in small water bodies, such as in recirculating aquaculture systems [8,9]. With the advancements in information technology, machine vision technology has gained popularity in aquaculture for mass estimation. Machine vision technology, a noninvasive, repeatable tool, offers an effective way to remotely monitor fish mass under different scenarios [10].

Two kinds of strategies for nonintrusive mass estimation are most commonly used in machine vision technology: parameter–weight prediction models and volume reconstruction. The parameters of the prediction model, such as body length, height, area, and perimeter, are extracted and then fitted using correlation analysis and regression analysis to establish a correlation model. For instance, a deep learning-based segmental analysis technique was developed by Abinaya et al. to determine the length characteristics of fish accurately and to establish a robust correlation between length and mass for the precise estimation of fish biomass, achieving an accuracy rate of 94.15% for the testing set and 91.52% for the validation image set [11]. Lu Zhang et al. extracted nine shape features (including area, perimeter, length, and width) from fish images to estimate fish mass using a principal component analysis–calibration factor (PCA-CF) method combined with a neural network. Their approach achieved a mean absolute error of 0.0104 and a coefficient of determination (R^2^) of 0.9021 on a carp dataset [12]. A multiparameter weight prediction model was developed by Hao et al. based on ventral geometrical features (area, length, and height), excluding the tail fin, using a non-supervised method. This approach enhanced the accuracy of fish quality prediction models, achieving an R^2^ of 0.991 [13]. A computer vision system that measured the body area, length, height, and eccentricity of Nile tilapia was designed by Fernandes et al. to predict fish mass and carcass mass using a linear model, achieving R^2^ values of 0.96 and 0.95 for body weight and carcass weight, respectively [14]. These studies primarily involve extracting two-dimensional feature parameters of fish to develop parameter–weight prediction models. However, this process requires restricting the fish within limited space and specific orientations (typically involving out-of-water procedures), which is challenging for measuring free-swimming fish [15,16].

To overcome these limitations, stereo vision technology based on binocular sensors has depth information available to measure the mass of freely swimming fish without confining the fish to a specific location [17]. Binocular vision captures the same target simultaneously using two cameras with the same parameter settings but in different positions, simulating the human eye perception of the real world [18]. This approach enables the reconstruction of the 3D coordinates of the fish by employing the principle of triangulation, which calculates the parallax of corresponding pixel points. The mass of swimming salmon was estimated by Tillett et al. using a pair of waterproof cameras, determining the length–weight relationship with a mean measurement error of 18% and a standard deviation (SD) of 9%. However, the approach required the manual identification of fish boundaries and key measurement points, making it time consuming and laborious [19]. A semiautomatic method was employed by Shafait et al. (2017) to obtain underwater stereo video for measuring the length of freely swimming Southern Bluefin Tuna (Thunnus maccoyii). This method calculated the mass using a length–weight equation derived from the average length of representative fish with an error margin of less than 1% against actual lengths. Although this method improved the estimation speed and reduced workload, it was not fully automated [20]. The fish body area was extracted by Shi et al. from freely swimming fish to estimate fish mass using stereo vision techniques and pattern recognition. The average relative errors between the estimated and measured values were 3.37% (orthogonal angle), 4.95% (greater than 45 angles), and 16.59% (less than 45 angles). The system required automatic search and selection of underwater real-time video using a fixed template image, making the processing relatively complex [15]. Although these methods have been proven effective and noninvasive for measuring the mass of freely swimming fish, they required complicated software for synchronizing cameras and handling complicated manual calibration procedures.

The alternative strategy for fish mass estimation is directly measuring their volume in water, which is simpler and more effective compared to establishing relational models. Accurately acquiring fish volume is a crucial parameter in this direct mass estimation method [21]. Laser scanning techniques were utilized by Almansa et al. to obtain volume measurements for estimating the biomass of a Senegalese sole population underwater. However, the use of time-of-flight (TOF)-based lidar for underwater target detection was limited by the transmitting lens aperture Rayleigh, and the laser device itself was large, heavy, costly, and not easily integrated into intensive farming systems [22]. However, this system required a precise alignment for the laser beam and was expensive and challenging to set up. Fish volume was estimated by Silva et al. using the point cloud generated by a stereo camera to estimate fish mass. However, only preliminary results were presented, and further experimental research was required to validate and improve the method [23].

In the context of this research, single-point 3D imaging technology, that is, an RGB-D sensor, which consists of a stereo depth module and vision processor, was utilized. An RGB-D sensor offers depth information with less computational and power consumption than other stereovision methods, which makes it an ideal sensor in siu for estimating fish mass noninvasively. Consumer-grade RGB-D cameras, known for their affordability, high frame rate, and quality, have found widespread application in harvesting robots. Some scholars have already tried to use RGB-D cameras for the estimation of fish mass. Microsoft Kinect was used by Saberioon and Cisar (2018) to estimate fish mass, achieving a remarkable R^2^ value of 0.872 [24]. Nevertheless, this was a preliminary experiment, and depth information was still affected by stray flashes. Moreover, only one species with eight dorsal geometrical features was reported to estimate the mass, which limited its generalizability across different fish species or sizes, potentially constraining its applicability.

In this paper, a noninvasive volume reconstruction model was proposed for the precise mass estimation of sturgeon based on RGB-D sensor. The volume of individual sturgeon was reconstructed by integrating the thickness of the upper surface of the sturgeon, and the difference in depth between the surface and the bottom was used as the thickness measurement. The method can utilize an RGB-D camera to obtain reliable, accurate measurement of the mass of sturgeon in real time. The strategy required no special hardware and intensive calculation, and it provided a key to uncovering noncontact, high-throughput, and highly sensitive mass evaluation of sturgeon while holding potential for evaluating the mass of other cultured fishes.

## 2. Materials and Methods

### 2.1. Overall Framework

**Figure 1:**
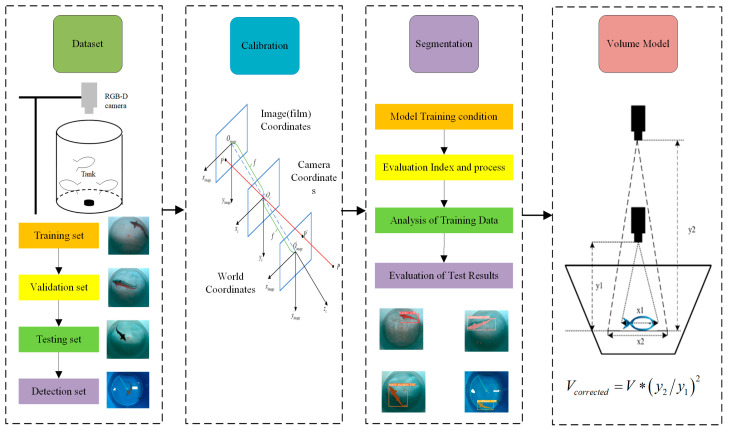
The overall framework of the nonintrusive volume reconstruction.

Figure 1 illustrates the overview scheme of the proposed volume reconstruction model of swimming sturgeon based on RGB-D sensor, which contains four parts. First, images were collected through data acquisition devices to build the dataset of our study, including a training set, validation set, testing set, and detection set. Second, the camera was calibrated to complete the transformation of the image-coordinate system into a camera-coordinate system and then into a world coordinate system to prepare for volume calculation in the succeeding steps. Simultaneously, instance segmentation was performed on the collected images using the YOLOv5s model, and the results of training and testing were evaluated. After achieving satisfactory accuracy, the sturgeon’s volume was reconstructed to ultimately obtain its weight.

### 2.2. Calibration

Because the RGB-D camera comprised two identical near-infrared cameras and one RGB camera, the calibration involved determining the internal parameters of the depth cameras and RGB sensor as well as the external parameters from the left near-infrared camera and the RGB camera.

#### 2.2.1. Calibration Devices

**Figure 2:**
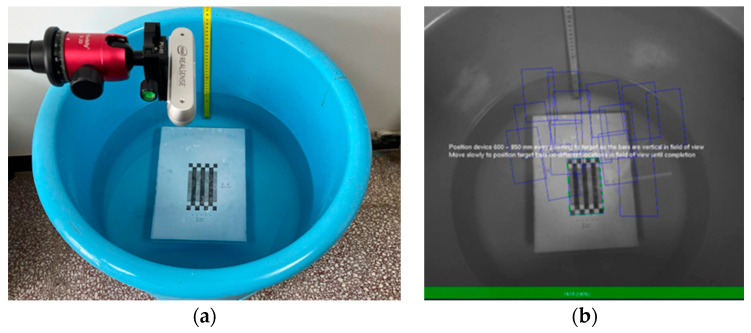
Calibration setup: (**a**) calibration target placed in the water, and (**b**) dynamic calibrator process with targeted calibration.

The official calibration tool provided by Intel, called Dynamic Calibrator, was utilized for the calibration of the depth and RGB cameras. The calibration setup is illustrated in Figure 2a, where a tripod was used to position the camera horizontally above the water surface. Before commencing calibration, the distance between the calibration plate and the depth camera was maintained between 60 and 85 cm. During the calibration, the depth camera was gradually moved, enabling the software to capture 15 images of the calibration plate from different positions, as shown in Figure 2b.

The calibration procedure for the RGB camera was the same as that for the depth camera. After collecting 15 images from different positions, the software can obtain all the calibrated parameters and save them in the camera.

#### 2.2.2. Aligning the Depth Frame to the Color Frame

**Figure 3:**
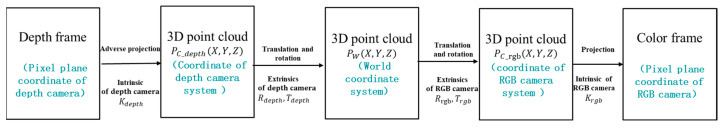
Process of aligning from the depth frame to the color frame.

After obtaining the internal parameter matrix and external parameter matrix of each camera, the depth and RGB frame must be registered to obtain the corresponding depth value of the pixel in the color image or the coordinate value in the world coordinate system. The flow chart of image registration is shown in Figure 3.

First, the depth frame of the pixel plane coordinate was adversely projected to the coordinate of the depth camera system through the intrinsic depth camera $K_{depth}$; therefore, the $P_{C\_depth}\left(X,Y,Z\right)$ of the corresponding space point in the depth camera coordinate system was calculated. Then, according to the external parameters $R_{depth}$ and $T_{depth}$ of the depth camera, the 3D point cloud $P_{w}\left(X,Y,Z\right)$ in the world coordinate system was calculated. Third, according to the external parameters $R_{rgb}$ and $T_{rgb}$ of the RGB camera, the point $P_{w}\left(X,Y,Z\right)$ was converted to the coordinate system of RGB camera $P_{C\_rgb}\left(X,Y,Z\right)$. Finally, according to the internal parameter $K_{rgb}$ of the RGB camera, the point $P_{C\_rgb}\left(X,Y,Z\right)$ was projected to the pixel plane coordinate of the RGB camera, i.e., the alignment from the depth frame to the color frame was completed.

### 2.3. Fish Segmentation

#### 2.3.1. Images Acquisition System and Dataset Creation

The image acquisition system used in the experiments consisted of an RGB-D camera and a Raspberry PI. For the purposes of this paper, the Intel RealSense D455 camera was selected after evaluating the performance of various RGB-D sensors. The Intel RealSense D455 camera employs active infrared stereo vision technology based on the principle of structured light, which enables the synchronous capturing of RGB and depth frames. This feature makes it suitable for indoor and outdoor applications, with the advantages of high resolution, extended range, compact size, and cost-effectiveness. This feature can be seamlessly integrated into intelligent aquaculture equipment. The RGB frame resolution, frame rate, and field of view were 1280 × 800, 30 and 90 × 65°, respectively. Depth output resolution, frame rate and field of view were 1280 × 720, 90 and 87° × 58°, respectively. The depth detection range of the camera was reliable within 0.6–6 m, with an error rate of less than 2% at a distance of 4 m (IntelRealSense, 2022). To minimize the reflection from the water surface, a polarizing film was added to the camera lens. The RGB-D camera was positioned vertically above the water surface, with a distance of 0.8–1.2m. The captured images were then sent to a computer for further processing.

The experiments were conducted in the aquaculture laboratory of the Institute of Fisheries Science, Beijing Academy of Agriculture and Forestry Sciences. To improve the generalization ability of the sturgeon segmentation model, two aspects were considered. First, the dataset utilized in this study encompassed sturgeon with diverse weights, sizes, distances, postures, colors, and species. Second, data-augmentation techniques were extensively used to further improve performance. These transformations included the adding of random pixel values, horizontal and vertical flips, 180-degree clockwise rotations, random rectangular occlusion, Gaussian noise, and random zeroing of pixels. We implemented these transformations using Python scripts in conjunction with the widely-used imgaug library, an open source tool for image enhancement extensively employed in machine learning and computer vision for image data augmentation. Table 1 shows the number of dataset images.

All images were divided into four datasets: training set, validation set, testing set, and detection set. The training set and validation set were used for model training, the testing set was used to test the performance of the segmentation of swimming sturgeon, and the detection set was used to detect the performance of the proposed volume-reconstruction model. In the experiment, the dataset was divided into training, validation, and testing sets in an 8:1:1 ratio. Different from the detection set that contained only images, the training set, validation set, and testing set contained images and corresponding annotation files in the .txt format. The annotation process was operated manually with Labelme v5.4.1.

The detection set was used to validate the performance of the proposed mass-estimation method, wherein three sets of experiments were conducted, each comprising 27 sturgeon The research focus of this study was *Acipenser ruthenus*, which were randomly selected from different breeding batches at the National Sturgeon Breeding Farm, covering an age range from three months to six years, for subsequent measurements. A total of 81 fish were collected. Each sturgeon was individually photographed and weighed before being returned to the breeding tank. The RGB and depth frames of each sturgeon were captured three times, and 60 frames were obtained during each capture session. Finally, a total of 14,580 sturgeon images were collected. Each sturgeon was individually photographed and weighed before being returned to the breeding tank. After image collection, each fish was caught and weighed three times as replicates.

#### 2.3.2. YOLOv5s Sturgeon Segmentation Algorithm

**Figure 4:**
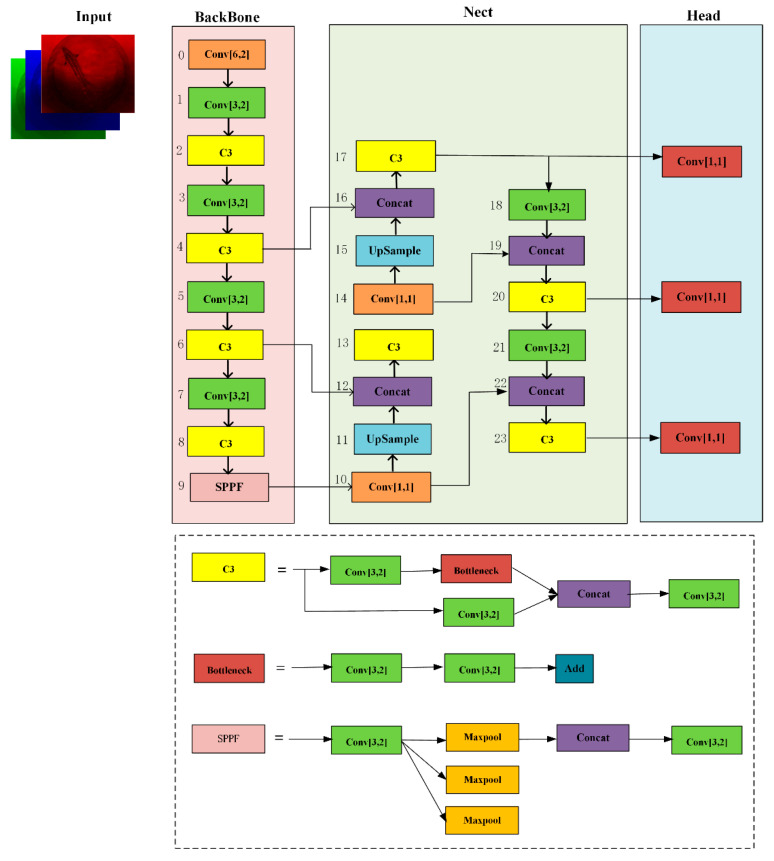
Schematic of the architecture structure of YOLOv5s algorithm. The entire network is divided into four parts: the input layers, the backbone feature extraction networks, the neck enhancement feature extraction networks, and the head layers.

To capture the features of sturgeon effectively and achieve accurate swimming sturgeon segmentation, the YOLOv5s algorithm was performed in the sturgeon segmentation task. The hierarchical network structure was divided into four parts: the input layers, the backbone feature extraction networks, the neck enhancement feature extraction networks, and the head layers, as shown in Figure 4.

The input layer in the YOLOv5s network processed the input images by applying techniques such as augmentation, adaptive anchor box computation, and adaptive image scaling. These methods can improve the generalization ability of the model and its training speed and accuracy. The preprocessed images are then fed into the backbone network, setting the foundation for accurate segmentation and detection of sturgeon masks and bounding boxes. The backbone feature-extraction networks extracted features from the input images by using a series of convolutional layers, including Conv, C3, and SPPF modules. This network processed the images to obtain features of sturgeon masks and bounding boxes at different levels, which were then passed into the neck for further fusion and enhancement, thereby ensuring robust segmentation and detection. The neck enhancement feature extraction networks in the YOLOv5s network were critical for sturgeon feature fusion and enhancement. They combined the feature pyramid network (FPN) and path aggregation network (PAN), also referred to as PANet. The FPN captured rich semantic features from higher layers, whereas the PAN aggregated positional information from lower layers. This dual-path approach improved the transmission of semantic and positional information, enhancing the network’s capability to detect sturgeon at multiple scales with high accuracy. The head layers were designed for multi-scale detection, extracting features from the third, fourth, and fifth layers to predict the positions and categories of sturgeon masks and bounding boxes. This design ensured the accurate and comprehensive segmentation and detection of sturgeon across various scales.

Model training was performed in a deep-learning environment with the following configuration: Windows11 system, Intel Gen Intel(R) Core (TM) i9-13900HX@2.20 GHz CPU, RAM 32 GB, NVIDIA GeForce RTX 4080 GPU. The integrated development environment was PyCharm 2020.3.5. The version of Python and Pytorch was 3.8 and 1.7, respectively. The parallel computer framework was CUDA 11.4.0, and the DL acceleration library was cuDNN 8.2.2.

In this study, the training parameter of the number of epochs, the batch size, and the learning rate were set at 100, 16, and 0.01, respectively. The Stochastic Gradient Descent with Momentum method was selected as the optimizer, with the momentum value being set at 0.937. To prevent overfitting the network, the weight decay was set at 0.0005. These settings ensured the accurate and gradual updating of network parameters during the learning process.

To evaluate the performance of a YOLOv5s sturgeon segmentation algorithm, the precision (*P*), recall (*R*), average precision (*mAP*), and *F*1 score served as evaluation metrics for the experiment. The calculation formulas were as follows:

(1) $$P=\frac{TP}{TP+FP}$$

(2) $$R=\frac{TP}{TP+FN}$$

(3) $$AP=\int_{0}^{1}\left(P\times R\right)dR$$

(4) $$mAP=\frac{1}{n}\sum_{i=1}^{n}AP_{i}$$

(5) $$F1-score=\frac{2\times P\times R}{P+R}$$

### 2.4. Estimation of Sturgeon Mass

#### 2.4.1. Reconstruction of Sturgeon Volume

**Figure 5:**
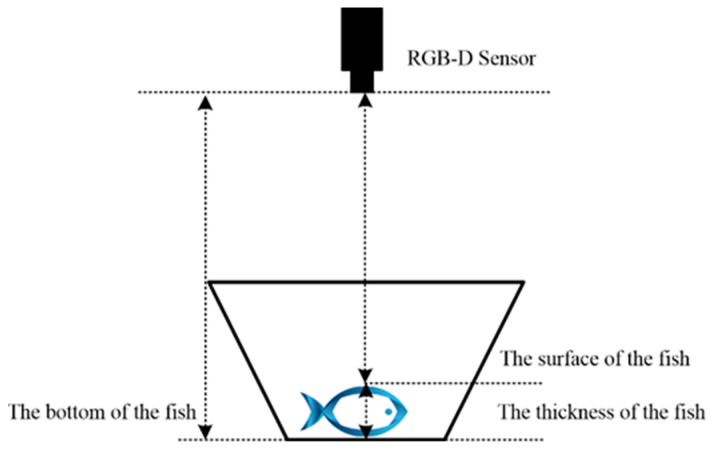
Schematic of sturgeon volume calculation using depth correction through RGB-D imaging. The figure illustrates the principle of reconstructing sturgeon volume by integrating pixel thickness along the fish’s body. Pixel thickness is determined by the difference between each pixel’s depth and the bottom of the fish.

The depth data acquired from the RGB-D sensor provided information on the distance from the camera to the upper surface of the fish and the distance from the camera to the bottom of the tank. However, the depth data did not directly provide the depth of the camera from the bottom of the fish, which was necessary for calculating the volume of the sturgeon accurately. To address this issue, the fish was assumed to be positioned close to the bottom of the tank, i.e, the bottom of the tank coincided with the bottom of the fish. However, the aquarium was a hemispherical object, and the depth values within it varied across different locations. Consequently, the most representative depth for the back of the fish was the depth of the tank at the edge of the fish. In this paper, a method to determine the depth of the bottom of the fish by averaging the depths of the pixels surrounding the fish’s body in a circular manner was proposed. By calculating the average depth around the circumference of the fish, the depth of the bottom of the fish was estimated. The volume of the sturgeon was computed by performing a cumulative integral of the thickness of each pixel along the fish’s body. The thickness of a pixel on the fish was determined by the difference between the depth of that pixel and the depth of the bottom of the fish, as shown in Figure 5.

#### 2.4.2. Corrected Volume Ratio Changes Caused by Lens Distance

**Figure 6:**
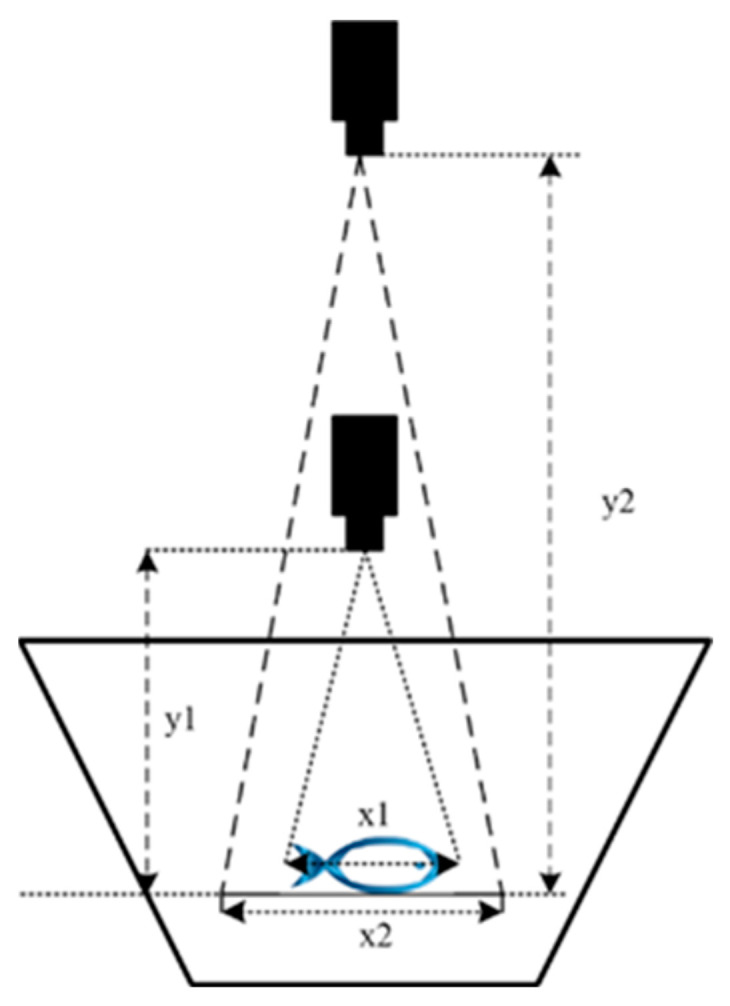
Dynamically calibrating sturgeon volumetric measurements considers swimming depth and lens-to-subject distance in sequential RGB-D imaging.

In the above principle of volume reconstruction of the sturgeon, the fish was assumed to be located at the bottom of the fish tank, but the fish was swimming freely in the water and its depth was not constant. Therefore, the volume needed to be corrected for the distance from the camera to the fish’s surface: $y_{1}$ and $y_{2}$ stand for the distance between the camera and the fish at the first measurement and second measurement, respectively, $x_{1}$ and $x_{2}$ stands for the width of the fish in the pixel plane at the first measurement and second measurement, respectively. Therefore, the second measurement was now corrected to the scale of the first, as shown in Figure 6.

Figure 6 shows that the proportional relationship among the different imaging processes conforms to the similar triangle property.

(6) $$x_{2}=x_{1}*y_{2}/y_{1}$$

W is the imaging field area, then

(7) $$W_{2}=W_{1}*{\left(y_{2}/y_{1}\right)}^{2}$$

S represents the surface area of fish, and S should be inversely proportional to W; then,

(8) $$S_{2}=S_{1}*{\left(y_{1}/y_{2}\right)}^{2}$$

V represents the volume of the fish. Given that its body thickness is constant for the same fish, if V is proportional to S, then

(9) $$V_{corrected}=V*{\left(y_{2}/y_{1}\right)}^{2}$$

Thus, the corrected fish volume can be determined and used to estimate the sturgeon mass.

## 3. Results

### 3.1. Calibration Results and Verification

Calibration was conducted following the methodology outlined in Section 2.2. The results of the calibrated camera parameters were as follows:

Intrinsic of depth camera $K_{depth}$:

(10) $$K_{depth}=\left[\begin{array}{ccc} 648.890 & 0 & 637.205\\ 0 & 648.890 & 365.610\\ 0 & 0 & 1 \end{array}\right]$$

Intrinsic of RGB camera $K_{rgb}$:

(11) $$K_{rgb}=\left[\begin{array}{ccc} 639.446 & 0 & 649.125\\ 0 & 638.697 & 372.294\\ 0 & 0 & 1 \end{array}\right]$$

Rotation from the RGB camera coordinate system to the left camera coordinate system $R_{rgb}$:

(12) $$R_{rgb}=\left[\begin{array}{ccc} 1.000 & 0.001 & 0.002\\ -0.001 & 1.000 & 0.0004\\ -0.002 & -0.0004 & 1.000 \end{array}\right]$$

Translation from the RGB camera coordinate system to the left camera coordinate system $T_{rgb}$:

(13) $$T_{rgb}=\left[\begin{array}{c} -59.157\\ -0.039\\ 0.0004 \end{array}\right]$$

Rotation from the right camera coordinate system to the left camera coordinate system $R_{rl}$:

(14) $$R_{rl}=\left[\begin{array}{ccc} 1.000 & 0.002 & -0.013\\ -0.002 & 1.000 & -0.004\\ 0.013 & 0.004 & 1.000 \end{array}\right]$$

Translation from the right camera coordinate system to the left camera coordinate system $T_{rl}$:

(15) $$T_{rl}=\left[\begin{array}{c} -95.074\\ 0.171\\ -0.521 \end{array}\right]$$

A high-precision alumina standard calibration plate (±0.01 mm) with a known checkerboard size of 50 mm was used for calibration examination. The calibration plate was positioned at various angles in 3D space, and the distance between adjacent corner points of two red dots on the checkerboard was measured using the depth camera to verify the actual distance. To achieve this, a calibration verification program was developed using PyCharm (PyCharm Community Edition) as the development environment. Fifty measurements were selected for verification, and the average measured value and absolute error were 50.013 and 0.013 mm, respectively, which closely matched the real value. The calibration performance was confirmed to be accurate within the acceptable range of measurement error.

### 3.2. Analysis of Model Training Results and Segmentation Test Results

**Figure 7:**
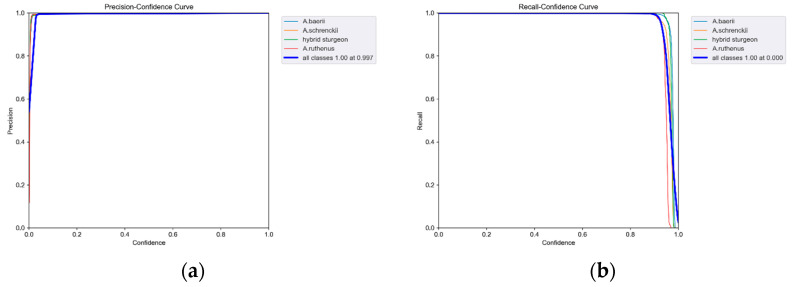

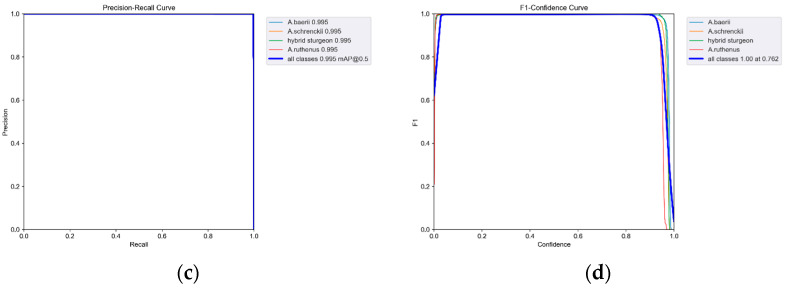
Training results of the YOLOv5s sturgeon segmentation algorithm: (**a**) precision–confidence curve; (**b**) recall–confidence curve; (**c**) precision–recall curve, and (**d**) F1–confidence curve.

To test the performance of the YOLOv5s algorithm in sturgeon segmentation, the curves of precision–confidence, recall–confidence, precision–recall, and F1–confidence were shown in Figure 7. It can be seen from Figure 7a that the precision reaches 0.989 at confidence levels of 0.03 for all classes of sturgeon. Figure 7b illustrates the relationship between recall and confidence for different sturgeon categories. It is evident that each sturgeon category maintains a recall above 0.9 across confidence levels ranging from 0 to approximately 0.91. This indicates that the recall rates for all sturgeon classes remain consistently high across a broad range of confidence levels. Since the area formed by the curves of different categories and the horizontal and vertical axes in the P-R curve represent the average precision of that category, it can be seen from Figure 7c that each category of sturgeon exhibits a significant area between the P-R curves and the X and Y axes. Furthermore, the equilibrium point is closer to the coordinate point (1,1), indicating that the YOLOv5s algorithm performed well in sturgeon segmentation. Figure 7d shows that the F1 score for all classes is 0.993 at confidence levels ranging from 0.025 to 0.913, indicating the model’s excellent balance of precision and recall in the above range of confidence.

**Figure 8:**
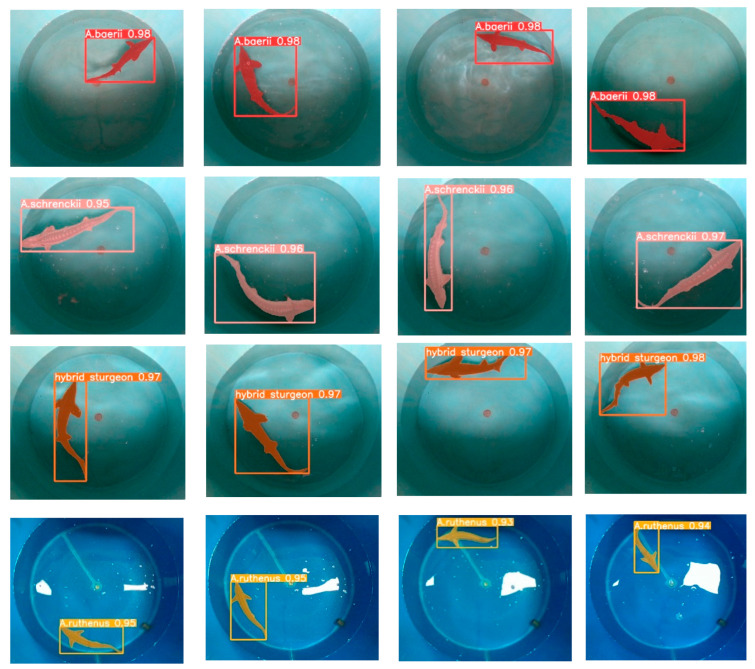
Examples of sturgeon segmentations of the Yolov5s algorithm with four species, i.e., a figure containing only one sturgeon to be detected. The bounding box shows the predicted label of each detected leaf and the confidence level of the prediction.

Examples of the detection effects of the YOLOv5s algorithm for sturgeon segmentations are shown in Figure 8. The detected sturgeon species include *A. baerii*, *A. schrenckii*, *hybrid*, and *A. ruthenus*, with confidence scores ranging from 0.93 to 0.98. Figure 8 demonstrates the ability to effectively detect and segment sturgeon across different species and conditions, including scenarios involving clear visibility as well as minor obstructions and variations in lighting. The precision, recall rate, and average value of the accuracy were shown in Table 2. Among them, the performance of all classes of sturgeon achieved an accuracy score of 0.88, a recall rate of 0.606, a mAP@0.5 score of 0.669, and a mAP@0.95 score of 0.521. Thus, it illustrates the strong performance of the YOLOv5s algorithm in sturgeon segmentation, as well as its high precision and recall rates across different conditions and sturgeon species.

### 3.3. Mass Estimation Results

**Figure 9:**
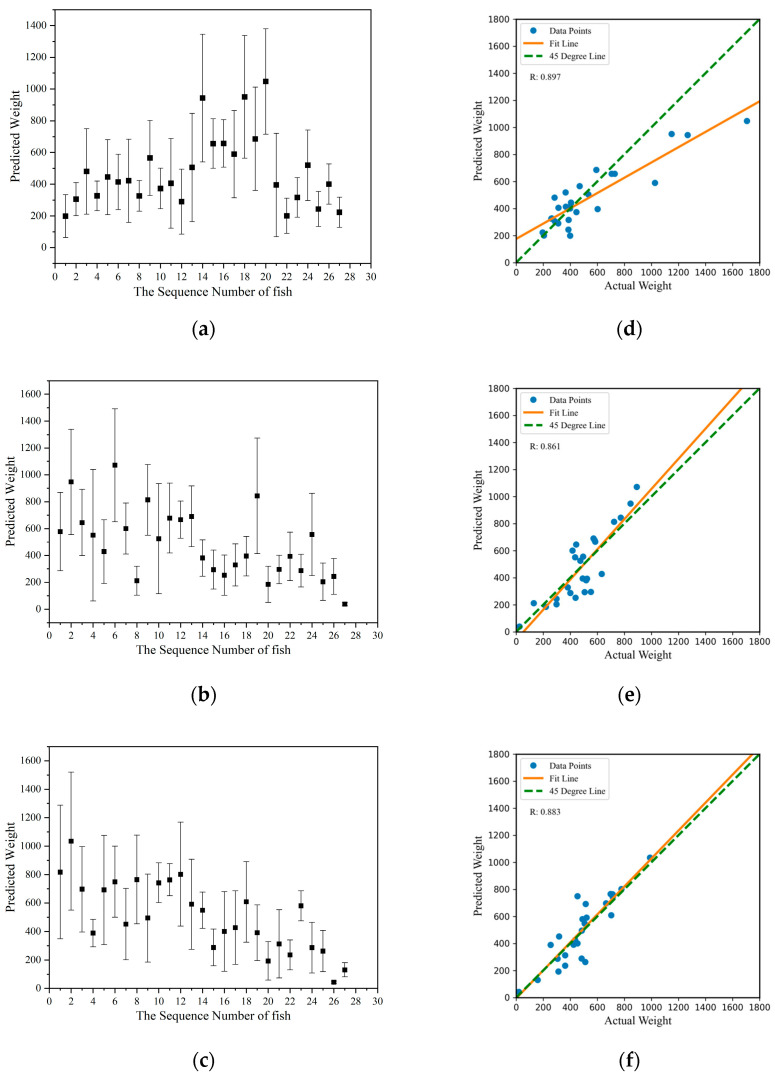
Predicted mass results of the detection set with three sturgeon groups (**a**–**c**) are presented for three different dates: 22 August 2022, 30 September 2022, and 17 November 2022, respectively. Each data point represents the arithmetic mean of individual sturgeon within each group, and 180 samples were obtained from three batches consisting of 60 frames each. The linear regression analyses of predicted versus actual masses for these groups were presented in (**d**–**f**).

In this paper, the mass of sturgeon was estimated using the detection set, which was gathered on three different dates: 22 August 2022, 30 September 2022, and 17 November 2022 (as shown in Figure 9a–c), respectively. Each data point represented the arithmetic mean of individual sturgeon within each group, and 180 samples were obtained from three batches consisting of 60 frames each. The error bar represented a 95% confidence interval, as shown in Figure 9a–c. The estimated masses ranged widely, from a minimum of 38.53 g in Group 2 to a maximum of 1071.23 g within the same group, which included the various sizes within the sturgeon population. The precision of these mass estimates was reflected in the variability of the SDs, capturing the range of uncertainty around the mean estimates in Figure 9a–c. The mean predicted mass, along with its SD for each group, was as follows: Group 1 had 477.23 g mean ± 211.31 SD, Group 2 had 485.35 g mean ± 221.14 SD, and Group 3 had 507.41g mean ± 223.34 SD, as shown in Table 3.

The validity of the proposed mass assessment method for all three groups of fish was assessed statistically using the Origin 2021 package. First, a Kolmogorov–Smirnov test was performed to determine the normality of the predicted mass data, and the results indicated that the predicted mass of the three groups of sturgeon followed a normal distribution. Subsequently, a one-way ANOVA was conducted to investigate the performance of the noncontact sturgeon mass assessment. The results indicated that the group means were not significantly different at the 0.05 significance level. Based on these findings, the proposed noncontact mass estimation method was consistent and reproducible.

The relationship between predicted and actual mass was further analyzed using linear regression, and the scatter plots in Figure 9d–f illustrate this comparison. Ideally, all points should align along the 45° line for perfect agreement. The R^2^ values were computed to evaluate the accuracy of these predictions. The results indicated an acceptable level of accuracy across all groups, as shown in Table 3. For example, Group 1 showed the highest prediction accuracy with an R^2^ of 0.897, suggesting a strong correlation between predicted and actual mass. Despite some outliers, the consistency of the R^2^ values across groups demonstrated the reliability of the method used for mass estimation.

**Figure 10:**
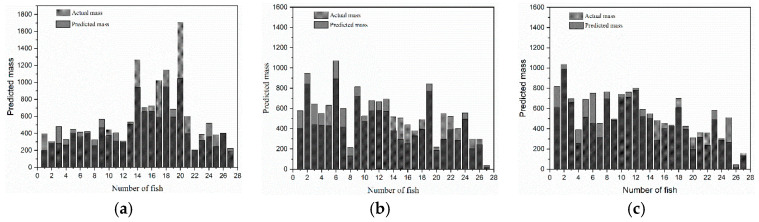
Predicted mass and actual mass of the three groups.(**a**) Group 1. (**b**) Group2. (**c**) Group3.

The actual masses of each sturgeon were compared with the corresponding masses obtained from the volume reconstruction model, and the results were plotted on a bar graph, as shown in Figure 10. In Group 1, 14 fish had predicted masses smaller than the measured masses, whereas 13 fish had predicted masses larger than the measured masses. In Group 2, 12 fish had smaller predicted masses, while 15 fish had larger predicted masses. Similarly, in Group 3, 11 fish had smaller predicted masses, while 16 fish had larger predicted masses.

To analyze further the cases where the predicted values were larger or smaller, all 81 fish were regrouped according to their true mass, as shown in Table 3. The table presents three sets: Set 1 with fish weighing less than 200 g, Set 2 with fish weighing between 200 g and 500 g, and Set 3 with fish weighing above 500 g. Among these sets, 80% of the fish had predicted values larger than the actual values in Set 1, as had 51% of the fish in Set 2 and 55% of the fish in Set 3.

To compare the influence of size on the accuracy of mass assessment quantitatively, further statistical analysis was performed on the experimental data. The 81 sturgeon were divided into three datasets based on their true mass. Similar to the previous method, a normality test using the Kolmogorov–Smirnov test indicated that the predicted mass in each dataset followed a normal distribution. Consequently, a one-way ANOVA revealed a significant difference between the datasets at the 0.05 significance level. Furthermore, the root mean square error (RMSE) was calculated for each dataset and to account for the different scales of the data. The RMSE was normalized by dividing it by the mean of the true mass. The results are presented in Table 4. Set 3 has the smallest normalized root mean square error (NRMSE), while Set 1 has the largest NRMSE.

## 4. Discussion

The present paper proposed a noninvasive volume reconstruction method for the precise mass estimation of sturgeon based on RGB-D sensor. Similar studies have been extensively reported [15,23,24]. This reliability was corroborated by other noninvasive fish mass estimation studies, supporting the validity of the technique used, as shown in Table 3.

To the best of our knowledge, this was the first paper to investigate nonintrusive mass estimation associated with volume reconstruction in sturgeon using RGB-D sensor. Studies on the nonintrusive mass estimation of other cultured fish species were previously reported. Laser scanning techniques were utilized by Almansa et al. to obtain volume measurements for estimating the biomass of a Senegalese sole population underwater [22]. However, the use of TOF-based lidar for estimating the biomass was limited by the low efficiency of imaging. Therefore, the method was only suitable for Senegalese sole, which swim very slowly. Moreover, the method exhibited overestimation in regard to low density or small fish in volume estimation. Therefore, in this paper, the measurement of the volume of sturgeon employed the RGB-D, which could obtain the depth information in real time. Fish volume was estimated by Silva et al. using the point cloud generated by a stereo camera to estimate fish mass [23]. However, the process involved calibrating a stereo camera, generating a disparity map with the Semi Global Block Matching algorithm, and creating a point cloud. Therefore, one limitation of the method was its reliance on the accuracy of the stereo camera calibration and the quality of the generated point cloud. The process was also sensitive to environmental conditions, such as lighting and water clarity, which may affect the quality of the stereo images and, consequently, the point cloud. This paper utilized an RGB-D camera with polarizing film. The strategy required no special hardware and intensive calculation, and it could minimize the reflection from the water surface. Saberioon and Cisar employed a Kinect RGB-D camera to capture the depth map and top view images of 295 seabass of varying sizes, from which the dorsal geometric features of fish were extracted for mass estimation [24]. However, the method’s reliance on specific dorsal geometrical features may limit its generalizability across different fish species or sizes, potentially constraining its applicability. In this paper, the volume reconstruction was based on the difference in thickness between the upper and lower surfaces, which offered greater generalizability to other fish species.

The volume of individual sturgeon was reconstructed by integrating the thickness of its upper surface, where the difference in depth between the surface and the bottom served as the measurement of thickness. This method enabled the reliable, accurate real-time mass estimation of sturgeon. The proposed strategy does not require specialized hardware or intensive calculations, and it offers a means to achieve noncontact, high-throughput, and highly sensitive mass evaluation of sturgeon. Furthermore, it holds potential for evaluating the mass of other cultured fish species.

One concern about the findings was the influence of size on mass estimation accuracy. Individual sturgeon within each group showed significant variability in mass estimates, marked by substantial SDs. This variability could be attributed to external factors such as disturbances on the water surface caused by fish movements. For example, the individual fish Number 15 in Group 1, Number 6 in Group 2, and Number 2 in Group 3 exhibited larger SDs in their mass estimates, as shown in Figure 9a–c. These results demonstrate that the accuracy of mass evaluation was significantly influenced by the size variation of the fish. The larger predicted values may be due to the segmentation being larger than the fish, caused by surface reflections, including splashes from fish movement and stray light from ambient lighting. Conversely, smaller predicted values could result from errors in eliminating redundant branches during image processing, leading to smaller fish segmentation. This result suggested that the proposed prediction method exhibited less error when evaluating larger fish, which may be attributed to the larger errors in image segmentation for smaller fish.

## 5. Conclusions

In this paper, a volume reconstruction model based on depth information for estimating sturgeon mass was presented. The proposed method offered accurate, noninvasive, and remote estimation of sturgeon mass. Unlike traditional 2D sensors, the RGB-D sensor used captured intensity and depth frames simultaneously, facilitating obtaining a foreground map of the sturgeon with depth information. By integrating the thickness on the upper surface of the sturgeon, its volume can be reconstructed. The statistical analysis results demonstrated that the proposed volume reconstruction model was consistent, repeatable, and satisfactory in estimating sturgeon mass. Compared with other nonintrusive estimation methods, our research directly obtained mass through volume calculation, resulting in lower computational requirements and power consumption. This approach provided inspiration for achieving noncontact, high-throughput, highly sensitive, and accurate evaluation of sturgeon mass. However, this paper focused solely on the model organism *A. ruthenus*. Future work should consider a wide range of sturgeon species to enhance the applicability and generalization of the proposed method further. Moreover, this paper did not consider the issues of fish occlusion.

## Figures and Tables

**Table 1:** The number of dataset images.

Image Data Set	Sturgeon Species	Number of Fish	Original Image	Augmentation	Total
Training set	*Acipenser baerii* *Acipenser schrenckii* *hybrid* *Acipenser ruthenus*	123	2040	2798	4838
Validation set	*Acipenser baerii* *Acipenser schrenckii* *hybrid* *Acipenser ruthenus*	123	254	350	604
Testing set	*Acipenser baerii* *Acipenser schrenckii* *hybrid* *Acipenser ruthenus*	123	254	350	604
Detection set	*Acipenser ruthenus*	81	14,580	-	14,580

**Table 2:** The detection accuracy of all classes of sturgeon with the YOLOV5s algorithm.

Classes	Precision (P)	Recall (R)	mAP@0.5	mAP@0.95
All	0.88	0.606	0.669	0.521
*A. baerii*	0.974	0.604	0.716	0.574
*A. schrenckii*	0.983	0.522	0.646	0.499
*hybrid*	0.834	0.583	0.634	0.531
*A. ruthenus*	0.728	0.714	0.679	0.478

**Table 3:** Predicted mass and standard deviation for three groups of sturgeon and the correlation coefficients of the accuracy of these predictions.

Group	Predicted Mass (g) ± Standard Devition (SD)	Correlation Coefficient (R^2^)
Group1	477.23 ± 211.31	0.897
Group2	485.35 ± 221.14	0.861
Group3	507.41 ± 223.34	0.883

**Table 4:** Predicted mass and standard deviation for three sets of sturgeon mass assessment.

Set	No.	RMSE	NRMSE
Set 1 (<200 g)	5	42.67	0.41
Set 2 (200 g–500 g)	43	115.12	0.30
Set 3 (>500 g)	33	195.84	0.27

## Data Availability

Data will be made available on request.
